# Supplementary material for: The role of maternal health care services as predictors of time to modern contraceptive use after childbirth in Northwest Ethiopia: Application of the shared frailty survival analysis
Source: PLoS One. 2020 Feb 4;15(2):e0228678. doi: 10.1371/journal.pone.0228678 (PMC6999900; doi:10.1371/journal.pone.0228678)
Supplement: S1 Table — (PDF) [file pone.0228678.s001.pdf]

Data Collection Tools _English Versions

I- Questionnaire for Kebele Administrators

	District and Community Characteristics			
	**Interviewer: This information need to be obtained from kebele administrators or formal delegate. Circle or fill in the blank space for responses according to the nature of the question**			
NO	QUESTION AND FILTERS		RESPONSE and CODE	SKIP
001	Questionnaire Id number		-----------------------	
002	Name of Woreda (District)		-----------------------	
003	Kebele Name			
004	Category of kebele		1. Urban 2. Rural	
005	Geographic location of the major part of the kebele		1. Dega<br>2. Woinadega<br>3. Kolla	
006	Total number of population in the kebele		____________________________	
007	What is the most usual transportation type for the Kebele's population to reach at the nearest<br>[ONLY ONE OPTION IS POSSIBLE]	Hospital	1. On foot<br>2. On mules/horseback<br>3. Vehicle<br>Others (Specify)…………	
		Health center	1. On foot<br>2. On mules/horseback<br>3. Vehicle<br>Others (Specify)…………	
		Health post	1. On foot<br>2. On mules/horseback<br>3. Vehicle<br>Others (Specify)…………	
009	What do you evaluate the nature of road to health facility?		1. Convenient<br>2. Inconvenient	
010	What is the average distance from center of kebele to main road		1. Near<br>2. Medium<br>3. Far	

II. Women's Questionnaire (English Version)

SECTION 1: ***Household*** **and Respondent's Background** ***Characteristics***

Interviewer : I am going to start by asking you some questions about you and your household<br>*Circle the answeramong alternatives OR fill in the blank space*			
Household Characteristics			
S.N	Question	Response and Code	Skip
101	How many family members are there in this house hold, including wife and husband? *Include only permanent residents(living greater than 6 months)*	Family members in number ________	
102	What is the main source of drinking water for members of your household?	1. Piped water<br>2. Dug well<br>3. Spring<br>4. River/stream<br>5. Others(*Specify*)____________	
103	Where is that water source located?	1. In own compound<br>2. Elsewhere	If in OWN compound go to 105
104	How long does it take to go there, get water, and come back?	Minutes................................<br>99. Don' t know	
105	What do you usually do to make the water safer to drink?<br>RECORD ALL MENTIONED	1. Nothing<br>2. Boiling<br>3. Add bleach/Chlorine<br>4. Filter through cloth<br>5. Others (Specify)…………..	
106	What kind of toilet facility do members of your household usually use?	1. No facility but bush/open field<br>2. Flush toilet<br>3. pit latrine<br>4. Other(specify)_________	
107	What type of fuel does your household mainly use for cooking?<br>[MULTIPLE OPTION POSSIBLE]	1. Electricity<br>2. Bio gas<br>3. Kerosene<br>4. Charcoal<br>5. Wood<br>6. Animal dung<br>7. Others (Specify)………………	
108	What type of fuel does your household mainly use for light source?<br>[MULTIPLE OPTION POSSIBLE]	1. Electricity<br>2. Bio gas<br>3. Kerosene lamp<br>4. Solar light<br>5. Others (Specify)………………	
109	Main material of the roof of main house<br>Record OBSERVATION	1. Corrugated iron sheet<br>2. Thatch/leaf	

<table>
<tr><td></td><td></td><td>3. Other(specify)_______</td><td></td></tr>
<tr><td>110</td><td>Main material of floor of main house is made of (observation)
Write ONLY ONE answer</td><td>1. Earth/ mud
2. ceramic tiles
3. cement
4. other [specify]_________________</td><td></td></tr>
<tr><td>111</td><td>Main material of the walls (observation)

Write ONLY ONE answer</td><td>1. wooden and mud
2. stone with lime/cement/ bricks
3. Wood plank
4. Other [specify]________________</td><td></td></tr>
<tr><td>112</td><td>What is now the primary source of income for this household?
Circle ONLY ONE answer</td><td>1. Farming, including livestock
2. employment/salary
3. petty trading (including sale of fire-wood, charcoal, grass etc)
4. Daily laborer
5. Other[specify]_________________</td><td></td></tr>
<tr><td>113</td><td>If the source of income is non farming, what is the estimated monthly income of the family in Birr [include all sources of income]</td><td>________________ Ethiopian Birr</td><td></td></tr>
<tr><td>114</td><td>How many of the following animals does this household own?
IF NONE, RECORD '00'.
[Probe and mark that all apply, multiple answer is possible]</td><td><table><tr><th>Animal type</th><th>Amount</th></tr><tr><td>Cows/oxen /Other cattle</td><td></td></tr><tr><td>Horses/donkeys/mules</td><td></td></tr><tr><td>Goats/Sheep</td><td></td></tr><tr><td>Chickens</td><td></td></tr><tr><td>Bee hives</td><td></td></tr></table></td><td></td></tr>
<tr><td>115</td><td>Does any member of this household own any agricultural land?</td><td>0. No
1. Yes</td><td>If No GO TO 117</td></tr>
<tr><td>116</td><td>How many hectares/or “Timad” of agricultural land do members of this household own? IF NONE, RECORD '00'.</td><td>Hectares__________ OR
“Timad”___________</td><td></td></tr>
<tr><td>117</td><td>Does any member of this household have an account with a bank/micro finance?</td><td>0. No
1. Yes</td><td></td></tr>
<tr><td>118</td><td>Is the house listed as model farmer?</td><td>0. No
1. Yes</td><td></td></tr>
<tr><td>119</td><td colspan="2"><table><tr><th colspan="2">Does your household have the following?</th><th>No (0)</th><th>Yes (1)</th></tr><tr><td>119.1</td><td>Radio/ Television</td><td></td><td></td></tr><tr><td>119.2</td><td>Telephone; landline/ mobile</td><td></td><td></td></tr><tr><td>119.3</td><td>Bed with cotton/sponges/spring mattress</td><td></td><td></td></tr><tr><td>119.4</td><td>An animal-drawn cart/Bicycle/motor Bike / Bajaj/ car?</td><td></td><td></td></tr><tr><td>119.5</td><td>Sofa/ chair with Arm or back rest</td><td></td><td></td></tr></table></td><td></td></tr>
</table>

Demographic and socio economic characteristics of respondents			
120	What is your age?<br>(WRITE ANSWER IN SPACE)	[_________________] years<br>OR<br>99. Don't Know	
121	Place of residence	1. Urban Area<br>2. Rural Area	
122	How long have you been living continuously in (name of current city, town or village of residence)?<br>IF LESS THAN ONE YEAR RECORD 'OO' YEAR	Years [_________]	
123	What is your current marital status?	1. Single/Never married<br>2. Married<br>3. Divorced<br>4. Widowed	**IF SINGLE GO TO 125**
124	At what age did you get marry?[include only the first marriage]	________________Years	
125	What is your current occupation?	1. Government Employee<br>2. Merchant<br>3. Farmer<br>4. Daily worker<br>5. Others (Specify)__________	
126	What is your partner/husband occupation?	1. Employee (GO/NGO)<br>2. Merchant<br>3. Farmer<br>4. Daily worker<br>5. Others (Specify)__________	
127	What is your religion?	1. Orthodox<br>2. Catholic<br>3. Protestant<br>4. Muslim<br>5. Others (Specify)	
128	What is your ethnicity?	1. Amhara<br>2. Agaw<br>3. Oromo<br>4. Others(specify) __________	
129	What is the highest GRADE you completed?	1. Cannot read and write<br>2. Read and write<br>3. Primary education<br>4. Secondary education<br>5. Higher education	
130	What is the educational level your partner/husband?	1. Cannot read and write<br>2. Read and write<br>3. Primary education<br>4. Secondary education<br>5. Higher education	

SECTION 2 - PRE-PREGNANCY, PRENATAL, INTRAPARTUM, AND POSTNATAL CARE<br>Interview Read: Now I would like to ask you some questions about your experiences with contraception, pregnancy childbirth and post-delivery care.			
No	QUESTION AND FILTERS	RESPONSE and CODE	
201	How many times have you been pregnant? (Gravida)	[_____] times	
202	How many live births have you had in your life? (Para)	[_________]livebirths:<br>________Male, ____Female	If she is Gravida I Skip Q#203
203	What is the interval between the birth of the last child and the birth of his/her immediate elder child	-------- (in months)	
204	Have you ever used any modern F/P method to delay or avoid getting pregnant of the last baby (NAME)?	1. No<br>2. Yes	
205	At the time you got pregnant with your last baby (NAME), did you want to get pregnant at that time?	0. No<br>1. Yes	IF YES GO TO208
206	If no to Q≠ 308, was your preference to become pregnant then or wait until later?	1. I wanted to be pregnant later<br>2. I didn't want to be pregnant then or any time in the future	
207	If your preference is to wait until later, how long did you prefer to wait?	1. Greater or equal to 24 months<br>2. Less than 24months<br>99. Don't Know………….	
	**Antenatal service utilization during the last Pregnancy**		
208	Have you ever attended ANC follow up for your current child? [ANC_ANY]	0. No<br>1. Yes	**IF NO GO TO 217**
209	During your most recent pregnancy, how many times did you access antenatal care?	[ ] times<br>OR<br>Don't remember …………………99	
210	How many months pregnant were you when you first accessed antenatal care for this pregnancy?	Months……………………… [___]<br>Don't remember …………………99	
211	Where did you get ANC service?	1. Government Hospital<br>2. Health center<br>3. Private clinic	

	[If there is referral linkage please, use the higher health care facility]	4. Home<br>5. Others(Specify)_______	
212	Whom did you see in your last ANC visit? Anyone else?<br><br>***PROBE TO IDENTIFY EACH TYPE OF PERSON AND RECORD THE ONE WHO SUPPORT AT LAST VISIT***	1. Doctor<br>2. Nurse<br>3. Midwife<br>4. Health officer<br>5. Health extension worker<br>99. I don't know<br>Other(Specify)_______________	
213	How long (on foot) it takes to reach at that health care facility. [RECORD THE LAST FACILITY IF VISITED MORE THAN ONE HEALTH INSTITUTE DURING ANC FOLLOW UP]	_________ Hours<br><br>I do not know…………99	
214	During (any of) your antenatal care visit(s), were you told about the signs of pregnancy complications or danger sign of pregnancy?	0. No<br>1. Yes	IF NO GO TO 216
215	Which signs of pregnancy complications were you told about?<br><br>*PLEASE DO NOT READ LOUD TO RESPONDANTS. PROBE TO TELL YOU MORE*<br>*MULTIPLE ANSWER IS POSSIBLE*	1. Vaginal bleeding<br>2. Vaginal gush of fluid<br>3. Severe headache<br>4. Blurred vision<br>5. Fever<br>6. Abdominal pain<br>7. Convulsion<br>Other (Specify) ……	
216	During any of antenatal visit, were you told about birth preparedness plan?	0. No<br>1. Yes	

Now, I would like to ask you about the overall experience of your *ANC* care when you were pregnant with (BABY'S NAME).As part of your antenatal care during this pregnancy, were any of the following done/happened by health providers at least once? (Put" ✓ "mark)

217		No(0)	Yes	DK(99)	
	body weight measured				
	Blood pressure measured				
	Urine sample taken				
	Tested for syphilis				
	advised about diet and nutrition during pregnancy				
	Counseling and testing of HIV/AIDS				
	Discussed the importance of F/ planning				
	Advantages of facility delivery and PNC				
	Discussed on adverse effect of malaria during pregnancy and its prevention(E.g ITN utilization)				

	**Labor and delivery**		
218	When did you give birth of your last baby	________/____/____	
219	How many months of gestation were for (BABY'S NAME) born?	………………….. Months<br>99. Don't know	
220	Where did you deliver (name of Baby)?<br>[PROBE TO IDENTIFY THE TYPE OF INSTITUTION]	1. Government Hospital<br>2. Government Health Center<br>3. Health Post<br>4. Private Hospital/Clinic<br>5. Home----------------------<br>99. Other(Specify:________)	If Home or others GOTO 223
221	What was the mode of delivery for your baby?	1. Spontaneous vaginal delivery<br>2. Assisted vaginal delivery<br>3. C/S	If spontaneous or Assisted GO TO 223
222	If delivered through C/S, did you have your cesarean operation plan before you went into labor or decided after your labor had already started?	1. Before labor started<br>2. After labor started<br>99.Don't know/can't remember	
	**Postnatal and new born care**		
223	Did you have any postnatal checkup in your last pregnancy?	0. No<br>1. Yes	IF NO GO TO 227
224	How long after the delivery did the FIRST health check take place?	1. Less than 24 hours<br>2. With in 25-48 hours<br>3. 49-72 hours<br>4. 73 hours-6 wks<br>5. > 6 weeks	
225	How many times you received the service.	________________Times<br>99. Donot know	
226	Where did you get the service	1. At own home<br>2. Health post<br>3. Health center<br>4. Public hospital<br>5. Private clinic/hospital<br>Other(Specify)……………	
	**For women who delivered at a health facility**		
227	How long you waited at the healthcare facility before discharged out?	…………. Hours	
228	After you gave birth to (NAME OF LAST CHILD), did anyone check on your health while you were still in the facility?	0. No<br>1. Yes<br>99. Do not know/Remember	
229	Did anyone check on your health after you left the facility?	0. No<br>1. Yes<br>99. Do not know	IF NO or DK GO TO232

230	How long after the delivery did the first check take place?***[Write in hours]***	…………… Hours		
231	Now I would like to ask you about the overall experience of your post-delivery care when you were pregnant with (BABY'S NAME). As part of immediate postnatal care, were any of the following done at least once? (Put " ✓ " mark)			
		No(0)	Yes(1)	
	Asked about the nature of vaginal discharge			
	Advised about breast feeding			
	Advised on diet and nutrition			
	Advised on family planning			
	Informed date of re-check up			
**Post-Partum Family planning**				
232	Since the birth of your child, has your menstruation resumed?	0. No<br>1. Yes		234
233	When, after birth of the child, does your menses resumed ?	_______________ weeks		
234	When did you start sexual intercourse after recent birth?	1. Not started yet<br>2. Within 7 days<br>3. 8-14days<br>4. 15-42 days<br>5. After 42 days		
235	Are you currently breasting feeding your child?	0. No<br>1. Yes		
236	Since birth of your last baby, were you visited by a health extension worker HEW) or did any staff member at the health facility counseled you on F/P?	0. No<br>1. Yes		
237	Since birth of your last baby, are you or your partner currently using any modern type of family planning?	0. No<br>1. Yes		**IF NO GO TO 240**
238	If yes to Q# 237, at what age of the last child did you /your partner start using the family planning method? ***Write in weeks***	Just at birth<br>OR<br>___________________<br>weeks		
239	Which method are you/your partner currently using? RECORD ALL MENTIONED	1. Female sterilization<br>2. Male sterilization<br>3. IUCD<br>4. Injectable<br>5. Implants<br>6. Pills<br>Others (specify)……………		
240	If no to Q# 237What was the reason for not using contraceptive.	1. Desire to have children<br>2. Low risk of pregnancy		

		3. Menstruation is not resumed<br>4. Husband or partner is not around<br>5. Opposition from partner<br>Other___________	
241	Does your husband support you in issues related to family planning services?	0. No<br>1. Yes	**IF NO GO TO 243**
242	How best does your husband assist you regarding the use of family planning services?	1. Taking F/P himself<br>2. Supporting through provision of transport<br>3. Reminding on dates of appointment<br>4. Gives items or finances<br>5. Use of condoms (both male and female condoms)<br>Any other (specify) …	
243	What is your future fertility preference?	1. Want next child Soon<br>2. Later than 24 months<br>3. Want no more<br>99. Unable to decide	
244	What is your pregnancy status now; pregnant, non- pregnant or do not know?	1. I am pregnant now<br>2. Not pregnant<br>99. Don't Know…………	
245	What is the ideal number of children you want to have[including the existing children] in your life	_____________	
Thank you for your information<br>Could you tell me please, any other information regarding this project? ---------------------------------			
