# Supplementary material for: The role of maternal health care services as predictors of time to modern contraceptive use after childbirth in Northwest Ethiopia: Application of the shared frailty survival analysis
Source: PLoS One. 2020 Feb 4;15(2):e0228678. doi: 10.1371/journal.pone.0228678 (PMC6999900; doi:10.1371/journal.pone.0228678)
Supplement: S2 Table — (PDF) [file pone.0228678.s002.pdf]

Data Collection Tools - Amharic Versions

አካባቢያዊ መረጃ

ይህን መጠይቅ ለመሙላት የቀበሌ አስተዳዳሪ/አመራር አካላትን ይጠይቁ።

<table>
<tr><th>ተ.ቁ</th><th colspan="2">መጠይቅ</th><th>መልስ</th><th>ማመልከቻ</th></tr>
<tr><td>001</td><td colspan="2">የጥያቄዉ መለያ ቁጥር</td><td>……………………..</td><td></td></tr>
<tr><td>002</td><td colspan="2">የወረዳዉ ስም</td><td>………………………</td><td></td></tr>
<tr><td>003</td><td colspan="2">የቀበሌዉ ስም</td><td>………………………</td><td></td></tr>
<tr><td>004</td><td colspan="2">ቀበሌዉ የሚገኝበት ቦታ</td><td>1 ከተማ
2 ገጠር</td><td></td></tr>
<tr><td>005</td><td colspan="2">የወረዳዉ አየር ፀባይ ሁኔታ</td><td>1- ደጋ
2- ወይናደጋ
3- ቆላ</td><td></td></tr>
<tr><td>006</td><td colspan="2">የቀበሌዉ ህዝብ ብዛት</td><td>……………..</td><td></td></tr>
<tr><td rowspan="3">007</td><td rowspan="3">ወደ አቅራቢያዉ ወደሚገኝ ተቋም(ሆስፒታል፤ጤናጣቢያ፤ጤና ኬላ)ለመጓጓዝ የሚጠቀሙበት የትራንስፖርት አይነት (አብዛናኛዉን ጊዜ የሚገለገሉበትን የትራንስፖርት አማራጭ ብቻ ይምረጡ)</td><td>ወደ ሆስፒታል</td><td>1 በእግር ጉዞ
2 በበቅሎ/ በፈረስ
3 በተሸከርካሪ
ሌላ (ይጠቀስ)……..</td><td rowspan="3"></td></tr>
<tr><td>ወደጤና ጣቢያ</td><td>1 በእግር ጉዞ
2 በበቅሎ/ በፈረስ
3 በተሸከርካሪ
ሌላ (ይጠቀስ)……….</td></tr>
<tr><td>ወደ ጤና ኬላ</td><td>1 በእግር ጉዞ
2 በበቅሎ/ በፈረስ
3 በተሸከርካሪ
ሌላ (ይጠቀስ)</td></tr>
<tr><td>008</td><td colspan="2">አምቡላንስን ጨምሮ ለድንገተኛ ጊዜ ነፍሰ-ጡር እናቶችን ወደ ጤና ተቋም ለማጓጓዝ ትራነስፖርት በተፈለገዉ ሰአት ይገኛልን;</td><td>1 አዎ ይገኛል
2 አይገኝም</td><td></td></tr>
<tr><td>009</td><td colspan="2">ወደ ጤና ተቋም የሚወስደዉን መንገድ እንዴት ይገመግሙታል;</td><td>1 አመች የሆነ
2 አመች ያልሆነ</td><td></td></tr>
<tr><td>010</td><td colspan="2">ከዚህ ቀበሌ እስከ ዋናዉ የመኪና መንገድ እርቀት እንደት የገልፁታል</td><td>1 ቅርብ
2 መካከለኛ
3 ሩቅ</td><td></td></tr>
</table>

የእናቶች ቃለ መጠይቅ

ክፍል አንድ፡ የቤትሁኔታ እና የቤተሰብ አባላትን በሚመለከት

ጠያቂ፡ ስለእርሰዎም ሆነ አጠቃላይ ስለቤተሰቡ ሁኔታእጠይቅዎታለሁ(መልሱን ያክብቡ ወይንም በተሰጠዉ ባዶ ቦታ ይሙሉ			
ተ.ቁ	መጠይቅ	አማራጭ	ቀጣይ ጥያቄ
101	በቤቱ ውስጥ ምን ያህል በቋሚነት የሚኖሩ የቤተሰብ አባላት ይኖራሉ?<br>*(ቋሚ አባላት የምንላቸዉ በአካባቢዉ ቢያንስ ለ6 ወራት የቆዩ ሲሆን ይህ ቁጥር ባልና ሚስትን የጨምራል)*	የቤተሰብ አባላት በቁጥር…….	
102	ቤተሰቡ በዋነኝነት ለመጠጥ ውሃ የሚያገኘው ከየት ነው?	1. ቧንቧ ውሃ<br>2. ጉድጓድ ውሃ<br>3. የምንጭ ውሃ<br>4. የወንዝ/የጅረት ውሃ<br>5.ሌላ(ይጠቀስ)<br>_______________	
103	ለመጠጥነት የምትጠቀሙበት ውሃ የሚገኝበት ቦታ የት ነው?	1. በግቢ ውስጥ--------------<br>2. ከግቢ ውጭ	105
104	ውሃ ከሚገኝበት ቦታ ቀድቶ ለመመለስ ምን ያህል ጊዜ ይወስዳል? በደቂቃ ይመዝግቡ	ደቂቃ................................<br>99. አላውቅም	
105	የተቀዳን ዉሃ ለመጠጥ ዝግጁ ለማድረግ በቤትዎ ዉስጥ ምን ዘዴ ይጠቀማሉ<br>*(በአብዛኛዉን ጊዜ የሚጠቀሙበትን አንድ አማራጭ ይጠቀሙ)*	1. ምንም አልጠቀምም<br>2. በማፍላት<br>3. ኬሚካል በመጨመር<br>4. በጨርቅ በማጥለል<br>ሌላ _______________	
106	ቤተሰቡ በዋናነት መፀዳጃ የሚጠቀመው የት ነው?	1. ሜዳ/በየቦታዉ<br>2. በውሃ የሚሰራ መፀዳጃ<br>3.የተለምዶ ጉድጓድ መፀዳጃ<br>4.ሌላ (ይገለፅ)____________	
107	ምግብ ለማብሰል የምትጠቀሙበት የሀይል ምንጭ ምንድን ነው?<br>*[ከአንድ በላይ አማራጭ መምረጥ ይቻላል]*	1. ኤሌክትሪክ<br>2. የተፈጥሮ ጋዝ<br>3. ነጭ ጋዝ<br>4. ከሰል<br>5. የማገዶ እንጨት<br>6. ኩበት<br>7.ሌላ__________________	
108	በቤት ዉስጥ ለብርሃን ምንጭነት የምትጠቀሙበት ዋና የሀይል ምንጭ ከየት ነው?	1. ኤሌክትሪክ<br>2. የተፈጥሮ ጋዝ<br>3. ኩራዝ	

<table>
<tr><td></td><td></td><td colspan="2">4.ሶላር(በፀሃይብርሃን
የሚሰራ)
5.ሌላ ______________</td><td></td></tr>
<tr><td>109</td><td>የመኖሪያ ቤቱ ጣራ የተሰራበት
ያዩትን ይመዝግቡ</td><td colspan="2">1. ቆርቆሮ
2. ሳር ክዳን
3. ሌላ______________</td><td></td></tr>
<tr><td>110</td><td>የቤቱ ወለል የተሰራበት
ያዩትን ይመዝግቡ (አንድ አማራጭ ብቻ ይምረጡ)</td><td colspan="2">1. አፈር/ጭቃ
2. ሴራሚክ
3. በሲሚንቶ/ሊሾ
4. ሌላ_____________</td><td></td></tr>
<tr><td>111</td><td>የቤቱ ግድግዳ የተሰራበት
ያዩትን ይመዝግቡ (አንድ አማራጭ ብቻ ይምረጡ)</td><td colspan="2">1 እነጨትና ጭቃ
2 ድንጋይ/ስሚንቶ/ጡብ
3 ሰምበሌጥ
4.ሌላ_________</td><td></td></tr>
<tr><td>112</td><td>ቤተሰቡ የሚተዳደርበት ዋና የገቢ ምንጭ የሆነዉ ምንድን ነዉ?</td><td colspan="2">1 ግብርና
2 በደሞዝ ተቀጣሪ
3.አነስተኛና ጥቃቅን ስራዎች
4.የቀን ሰራተኛ
5.ሌላ_______________</td><td></td></tr>
<tr><td>113</td><td>መተዳደሪዎ ከግብርና ዉጭ ከሆን የወር ገቢ ምን ያክል ነዉ?</td><td colspan="2">ብር……………</td><td></td></tr>
<tr><td rowspan="6">114</td><td rowspan="6">ከሚከተለት እንስሳት ውስጥ በቤት ውስጥ ምን ያህል አለ?
የተጠቀሱት የእንሰሳት አይነት በቤት ዉስጥ ከሌሉ “ 00” ብለዉ ይሙሉ
(ከአንድ አማራጭ በላይ መምረጥ ይቻላል)</td><td>የእንስሳት ዓይነት</td><td>ቁጥር</td><td rowspan="6"></td></tr>
<tr><td>ላም/ በሬ</td><td>…………</td></tr>
<tr><td>ፈረስ፤አህያ እና በቅሎ</td><td>…………</td></tr>
<tr><td>በግና ፍየል</td><td>…………</td></tr>
<tr><td>ዶሮ</td><td>…………</td></tr>
<tr><td>የንብ ቀፎ</td><td>…………</td></tr>
<tr><td>115</td><td>ቤተሰቡ ለእርሻ የሚሆን መሬት አለው?</td><td colspan="2">0 የለም-----------------------
1 አዎ</td><td>117</td></tr>
<tr><td>116</td><td>ለእርሻ የሚሆነው ቤተሰብ መሬት ስፋት ምን ያህል ካሬ/ጥማድ ይሆናል?</td><td colspan="2">ካሬ ሜትር…………
ወይንም
ጥማድ…………..</td><td></td></tr>
<tr><td>117</td><td>አሁን አብረዉ ከሚኖሩት የቤተሰቡ አባላት ውስጥ የባንክ ደብተር/አካውንት ያለው አለ?</td><td colspan="2">0 የለም
1 አዎ አለ</td><td></td></tr>
<tr><td>118</td><td>ቤተሰቡ በሞዴል አርሶአደርነት ተመዝግቧል?</td><td colspan="2">0- ሞዴል አርሶ አደር አይደለም
1-አዎ ሞዴል አርሶ አደር ነዉ</td><td></td></tr>
</table>

119	ከሚከተሉት እቃወች/ንብረቶች የትኞቹ በቤትሽ ውስጥ ይገኛሉ?	የለም (0)	አዎ (1)	
119.1	ራድዮ/ቴሌቪሽን			
119.2	ስልክ(ተንቀሳቃሽ/የመስመር)			
119.3	አልጋና ፍራሽ-ስፖንጅ/ጥጥ/ስፕሪንግ			
119.4	ጋሪ/ሞተር ሳይክል/መኪና/ባጃጅ			
119.5	ሶፋ/ የጀርባና እጅ መደገፊያ ያለዉ የእንጨት ወንበር			

የማህበራዊ ኢኮኖሚያዊ እና ስነህዝባዊ ባህሪያት

ቁጥር	ጥያቄ	መልስ	ማመልከቻ
120	ዕድሜ	ዓመት..............<br>99. አላዉቅም	
121	የሚኖሩበት ቦታ	1 ከተማ<br>2 ገጠር	
122	በዚህ አሁን በሚኖሩበት ቀበሌ ምን ያህል ጊዜ ቆዩ? *ከአንድ አመት በታች ከሆነ "00" ብለዉ ይመዝግቡ*	ዓመት	
123	የጋብቻዎ ሁኔታ<br>*(አሁን ያለዉን የጋብቻዎ ሁኔታ ይመዝግቡ)*	1. አላገባሁም/ብቸኛ ነኝ----<br>2. አግብቻለሁ<br>3. ፈትቻለሁ<br>4. ባሌ ሞቷሌ	125
124	በስንት አመትዎ ባል አገቡ<br>*(ይህ የመጀመሪያ ጋብቻን ብቻ የሚመለከት ይሆናል)*	ዓመት..............	
125	እርሰዎ የሚሰሩት ስራ ምንድን ነው?	1. የመንግስት ሰራተኛ<br>2. ነጋዴ<br>3. ግብርና<br>4. የቀን ሰራተኛ<br>ሌላ (ይጠቀስ)----------------	
126	ባለቤትዎ የሚሰሩት ስራ ምንድን ነው?	1. የመንግስት ሰራተኛ<br>2. ነጋዴ<br>3. ግብርና<br>4. የቀን ሰራተኛ<br>ሌላ (ይጠቀስ)----------------	
127	የየተኛዉ ሃይማኖት ተከታይ ነዎት?	1. ኦርቶደክስ<br>2. ካቶሊክ<br>3. ፕሮቴስታንት<br>4. ሙስሊም<br>5. ሌላ (ይጠቀስ) _______	
128	ብሄርዎ ምንዴነው?	1. አማራ<br>2. አገው<br>3. ኦሮሞ	

		4. ሌላ (ይጠቀስ) _______	
129	የትምህርት ደረጃ	1. አልተማርኩም<br>2. ማንበብናመጻፍ<br>3. አንደኛ ደረጃ(1-8)<br>4. ሁለተኛ ደረጃ (9-12)<br>4. ከፍተኛ ት/ት ተቋም	
130	የባለቤትዎ/ጓደኛዎ የትምህርት ደረጃ?	1. አልተማርኩም<br>2. /ማንበብናመጻፍ/<br>3. አንደኛ ደረጃ(1-8)<br>3. ሁለተኛ ደረጃ (9-12)<br>4. ከፍተኛ ት/ት ተቋም	

ክፍል 2: የእርግዝና፣ ወሊድና ፣ድህረ- ወሊድ ጤና አገልግሎት አጠቃቀምን በተመለከተ ***ጥያቄዎን እንደሚከተለዉ ይጀምሩ፣አሁን ከወሊድ በፊት፣ በወሊድ ወቅት እና ከወሊድ በኋላ ስለነበሩ ሁኔታወች አንዳንድ ጥያቄወችን ልጠይቅወት እፈልጋለሁ፡፡***

**የቅድመ- እርግዝና እና ስነተዋልዶ ታሪክ**			
ተ.ቁ	መጠይቅ	መልስ	
201	እስካሁን ድረስ ለምን ያህል ጊዜ አርግዘዋል? ***(ውርጃንና በወሊድ ጊዜ የሞተን ጨምሮ)***	----------------(በቁጥር ይቀመጥ)	
202	የመጨረሻውን ልጅዎን ጨምሮ ምን ያህል ሌጆችን በሕይወት ወልደዋል?	----------------(በቁጥር ይቀመጥ)	***ይህ የመጀመሪያ ልጅ ከሆነ ጥያቄ ቁጥር203 ይለፉ***
203	በቀድሞው ልጅዎና በአሁኑ ልጅዎ መካከል ምን ያህል የእድሜ ልዩነት አለዉ?	-----------አመት	
204	የቤተሰብ ምጣኔ ተጠቅመዉ ያዉቃሉ?	0. ተጠቅሜ አላዉቅም<br>1. አዎ ተጠቅሜ አውቃለሁ	
205	የመጨረሻ እርግዘናዎ **[የሕፃኑ ስም]** በወቅቱ የታሰበና/ የታቀደ ነበር?	0 የታቀደ አልነበረም<br>1 አዎ የታቀደ ነበረ………………..	208
206	የመጨረሻ እርግዘናዎ በወቅቱ የታሰበና/ የታቀደ ካልነበር እርግዝናዎ መቸ እንዲሆን ነበር የፈለጉ	1.እንዲዘገይ<br>2. ጭራሽ መዉለድ አልፈልግም ነበር	
207	የእርስዎ ምርጫ እንዲዘገይ ከነበር ለምን ያህል ጊዜ ነበር?	1 ሁለት አመት እና በላይ<br>2 ከሁለት አመት ያለበለጠ<br>99. አላዉቅም	
**የቅድመ- ወሊድ(የእርግዝና0 ክትትልን በሚመለከት**			
208	ይህንን ህፃን (ስም) ሲያረግዙ የእርግዝና ክትትል አድርገው ነበር?	0. አይደለም---------------------------<br>1. አዎ	217

209	ለምን ያህል ጊዜ ክትትል አደረጉ?	ጊዜ......................<br>99. አላስታውስም	
210	የመጀመሪያውን ክትትል ያደረጉት እርግዝናዎ ስንት ወር ሲሞላወት ነበር?	ወር.....................<br>99. አላውቅም/አላስታውስም	
211	የእርግዝና ክትትልን ያደረጉት የት ቦታ ነበር?	1. መንግስት ሆስፒታል<br>2. መንግስት ጤና ጣቢያ<br>3. ከግል ክሊኒክ<br>4. ቤት ውስጥ<br>5. ሌላ _______________	
212	የመጨረሻውን ክትትል ሲያደርጉ ምርመራ ያደረገልዎት ምን ዓይነት ባለሞያ ነበር?	1. ዶክተር<br>2. ጤና መኮነን<br>3. ነርስ/አዋላጅ ነርስ<br>4. የጤና ኤክስቴንሽን ባለሞያ<br>ሌላ (ይጠቀስ)______________<br>99. አላውቅም	
213	ጤና ተቋም ለመድረስ በእግር ጉዞ ስንት ደቂቃ ይወስዳል?	ደቂቃ ....<br>99. አላውቅም	
214	የእርግዝና ክትትል በሚያደርጉበት ወቅት በእርግዝና ወቅት ስለሚከሰቱ አደገኛ ምልክቶች ከጤና ባለሙያወች ተነገሩዎት ነበር?	0. አይደለም.......................<br>1. አዎ	216
215	ስለየትኛዎች አደገኛ ምልክቶች ከጤና ባለሙያወች ተነገሩዎት ? ***ከአንድ በላይ መልስ መምረጥይቻላል***	1. ከማህፀን ደም መፍሰስ<br>2. ምጥ ከመጀመሩ በፊት እንሽርት ዉሃ መፍሰስ<br>3. ከፍተኛ የራስ ምታትና<br>4. ብዥያለ እይታ<br>***5.*** ትኩሳት ***(የሰዉነትሙቀት መጨመር)***<br>6. የሆድ ህመም<br>7. የሰዉነት መንዘፍዘፍ/ራስንመሳት<br>ሌላ(ይጠቀስ)............	
216	በአለፈዉ የእርግዝና ክትትል ወቅት ስለወሊድ ቅድመ ዝግጅት እቅድ ማድርገ እንዳለብዎት ከባለሙያ ጋር ተነጋግረዋል	0. አይደለም<br>1. አዎ	

217	አሁን ይህንን ህፃን (ስም) ሲያረግዙ የነበረወትን ሁሉንም ክትትልች ያስታውሱ ። በነዚህ ክትትልች ወቅት የጤና ባለሞያወች -----('X' ምሌክት ያድርጉ)			
		የለም(0)	አዎ(1)	አላስታዉስም(99)
	ክብደትዎን መዘኑ?			
	የደም ግፊት(በክንድ ላይ በሚታሰር መለኪያ) እየለኩ ክትትል አደረጉ?			
	የሽንት ናሙና ወሰዱ?			
	የቂጥኝ/ዉርዴ ምርመራ አደረጉ?			
	በእርግዝና ወቅት ስለሚደረግ አመጋገብ ምክር ሰጡዎት?			
	ስለ ኤች አይ ቪ ኤድስ መከላከያና ምርመራ ተነጋገራችሁ?			
	ስለቤተሰብ ምጣኔ ተወያያችሁ?			
	ጤና ድርጅት መዉለድና ድህረ-ወሊድ አገልግሎት ማግኘት ስለሚሰጠዉ ጠቀሜታ ተወያችሁ?			
	ወባ በእርግዝና ወቅት ስለሚያስከትለው አደጋና እንዴት መከላከል እንደሚቻል (ምሳሌ አልጋ አጎበር በመጠቀም) ተወያያችሁ?			
	**የወሊድ አገልግሎትን በተመለከተ**			
218	ህፃኑ(ስም) የተወለደበት ቀን፤ ወር፡ዓ.ም	_________/_____/_____		
219	ይህንን ህፃን (ስም) ሲወልዱ እርግዝናው የስንት ወር ነበር?	ወር-----------<br>99. አላውቅም		
220	ህፃኑ(ስም) የወለዱበት ቦታ የት ነበር?	1. የመንግስት ሆስፒታል<br>2. የመንግስት ጤና ጣቢያ<br>3. ጤና ኬላ<br>4. የግል ሆስፒታል/ክሊኒክ<br>5. ቤት<br>ሌላ (ይጠቀስ)…………….		
221	ህፃኑ(ስም)ሲወልዱ የዉልደት ሁኔታ እንዴት ነበር? በራሱ በተፈጥሮ አምጦ	1. በተፈጥሮ አምጦ በመዉለድ----<br>2. በኦፕራሲኦን(ቀዶ ጥገና) ------<br>3. በመሳሪያ በመታገዝ<br>ሌላ(ይገለጽ)_____________		223<br>223

	በመዉለድ፧በኦፕራሲኦን ወይንስ በመሳሪ በመታገዝ		
222	የወለዱት በኦፕራሲኦን ከሆነ ዉሳኔዉ የተወሰነ ምጥ ከመጀመሩ በፊት ነዉ ወይንስ በኂላ ነበር	1. ምጥ ከመጀመሩ በፊት<br>2. ምጥ ከጀመረ በኂላ<br>99.አላውቅም………	
	**የድህረ ወሊድና ጨቅላ ህጻናት ጤና አገልግሎት**		
223	ህፃኑን (ስም) ከወለዱ በኂላ በባለሙያ የድህረ ወሊድ ምርመራ አድርገዋል?	1. አላደረግሁም----------------------<br>2. አድርጊያለሁ	227
224	ከወሊዱ ከስንት ቀን በኂላ የመጀመሪያውን ድህረ ወሊድ ምርመራ ፈፀሙ?	1. ከ 24 ሰአት ያነሰ<br>2. 25- 48 ሰአት<br>3. 49-72 ሰአት<br>4. 73ሰአት- 6 ሳምንት<br>5. ከ 6 ሳምንት በላይ	
225	ህፃኑን (ስም) ከወለዱ በኂላ በአጠቃላይ ለስንት ጊዜ ምርመራ ተደረገልዎ	………………<br>99. አላስታዉስም	
226	ህፃኑን (ስም) ከወለዱ በኂላ የመጀመሪያውን የድሀረ-ወሊድ አገልግሎቱን የት ቦታ ወሰዱ	1. መኖሪያ ቤት<br>2. ጤና ኬላ<br>3. ጤና ጣቢያ<br>4. ሆስፒታል<br>5. የግል ክሊኒክ/ሆስፒታል<br>ሌላ(ይጠቀስ)………..	
	**በጤና ድርጅት ብቻ ለወለዱት የሚጠየቅ**		
227	ህፃኑን (ስም) ከወለዱ በኂላ በጤና ተቋሙ ዉስጥ ለስንት ጊዜ ቆዩ? *መልሱ በሰዓት ይቀመጥ*	……………. ሰዓት	
228	ህፃኑን (ስም) ከወለዱ በኂላ በጤና ተቋሙ ዉስጥ እንዳሉ የጤና ባለሙ ምርመራ አድርጎልዎታል?	0. አልተደረገልኝም<br>1. ተደርጎልኛል<br>99. አላስታዉስም	
229	ህፃኑን (ስም) ከወለዱና ወደ ቤትዎ ከተመለሱ በኂላ በጤና ባለሙ ምርመራ አድርጎልዎታል?	0. አልተደረገልኝም------------------<br>1. ተደርጎልኛል<br>99. አላስታዉስም----------------------	232<br><br>232
230	ህፃኑን (ስም) ከወለዱና ወደ ቤትዎ ከተመለሱ በኂላ በጤና ባለሙ ምርመራ የተደረገልዎ ከስንት ጊዜ በኂላ ነዉ? ***በቀን ይፃፍ***	በ………… ቀን ዉስጥ	

231	አሁን ህፃን (ስም) ከወለዱ በኋላ በድህረ ወሊድ ምረመራ የተሰጠወትን ሁሉንም አገልግሎቶች ያስታውሱ ፡፡ በድህረ ወሊድ ምርመራ ወቅት ባለሞያወች ----- (‘X‘ ምልክት ያድርጉ)			
		የለም(0)	አዎ(1)	አላስታውስም<br>/አላውቅም(99
	ስለ ማህፀን ፈሳሽ ጠየቁዎት?			
	ስለ ጡት ማጥባት ምክር ሰጡዎት?			
	ስለሚወስዱት ምግብ ምክር ሰጡዎት?			
	ስለጡት ቤተሰብ ምጣኔ ምክር ሰጡዎት?			
	የቀጠሮ ጊዜ አሳወቅዎት?			
	**የድህረ- ወሊድ የቤተሰብ ምጣኔ አጠቃቀም**			
232	ህፃኑ (ስም) ከተወለደ በኋላ የወር አበባ ማየት ጀምረዋል?	0. ማየት አልጀመርኩም-----------<br>1. ማየት ጀምሬአለሁ		234
233	የወር አበባ ማየት ከጀመሩ ህፃኑ (ስም) ከተወለደ ከስንት ወር/ሳምንት በኋላ ማየት ጀምሩ? ***ወሩ ወደ ሳምንት ይቀየር***	ሳምንት -----------		
234	ህፃኑ (ስም) ከተወለደ በኋላ የመጀመሪያ ግብረስጋ ግንኙነት ማድረግ የጀመሩበት ጊዜ	1. ግንኙነት አላደረግሁም<br>2. በሳምነት ዉስጥ<br>3. ከ 8-14 ቀን ዉስጥ<br>4. ከ15-42 ዉስጥ<br>5. ከ 42 ቀናት በኋላ		
235	አሁን ህፃኑን (ስም) ጡት ያጠባሉ?	0. አላጠባም<br>1. አጠባለሁ		
236	ህፃኑ (ስም) ከወለዱ በኋላ የቤተሰብ ምጣኔን በተመለከተ በጤና አክስቴንሽን ወይንም በሌላ የጤና ባለሙያ የምክር አገልግሎት አግኝተዋል?	0. የለም<br>1. አዎ		
237	ህፃኑ (ስም) ከወለዱ በኋላ እርስዎ ወይንም ባለቤትዎ የቤተሰብ ምጣኔን መዉሰድ ጀምረዋል?	0. የለም-----------------------------<br>1. አዎ		240
238	ለጥያቄ ቁጥር 237 መልስዎ አዎ ከሆነ የቤተሰብ ምጣኔ መዉሰድ የጀመሩበት ጊዜ መቸ ነበር?**(ወሩን ወደ ሳምንት ቀይረዉ ይፃፍ)**	1. ከወለድሁበት ቀን ጀምሮ ወይንም<br>ሳምንት -----------		

239	እርስዎ ወይንም ባለቤትዎ እየወሰዱት ያለዉ የቤተሰብ ምጣኔ አይነት የትኛዉ ነዉ?	1. የሴት የእንቁላል ማስተላለፊያ ቱቦን በመዝጋት(በቀዶ ጥገና)<br>2. ወንድን የዘር ፍሬ ማስተላለፊያ ቱቦን በመዝጋት (በቀዶ ጥገና)<br>3. በማህፀን ዉስጥ የሚገባ (ሉፕ)<br>4. በየሶስት ወር በመርፌ የሚሰጥ(ዲፖ)<br>5. በክንድ የሚቀበር(ኢምፕላኖን)<br>6. በአፍ የሚዋጥ(እንክብል)<br>ሌላ (ይጠቀስ)……………………	
240	የወሊድ መከላከያ የማይወስዱ ከሆነ ያልወሰዱበት ምክነያት ምንድን ነዉ?	1. ሌላ ልጅ መዉለድ ስለምፈልግ<br>2. በዚህ ወቅት አላረግዝም ብየ ስለማስብ<br>3. የወር አበባ ማየት ስላልጀመርኩ<br>4. ባለቤቴ አብሮኝ ስለማይኖር<br>5. ባለቤቴ ስለማይፈቅድ<br>ሌላ (ይጠቀስ)……………………	
241	ባለቤትዎ የእርስዎን የቤተሰብ ምጣኔ መዉሰድ ይደግፋሉ ?	0. አይደግፉም-------------<br>1. አዎ፣ ይደግፋሉ	243
242	ባለቤትዎ የእርስዎን የቤተሰብ ምጣኔ መዉሰድ የሚደግፉት በምን መልኩ ነዉ	1. የቤተሰብ ምጣኔ በመዉሰድ መሳተፍ<br>2. ትራንስፖርት በማመቻቸት<br>3. የቀጠሮ ቀንን በማስታወስ<br>4. የገንዘብ ወይንም የቁስአካል ድጋፍ<br>ሌላ (ይጠቀስ)……………………	
243	ህፃኑ (ስም) ከወለዱ በኋላ ሌላ ልጅ የመዉለድ ፍላጎትዎ ይምን ይመስላል?	1. በአጭር ጊዜ ዉስጥ መዉለድ እፈልጋለሁ<br>2. መዉለድ የምፈልገዉ ከሁለት አመት በኋላ ነዉ<br>3. ጭራሽ መዉለድ አልፈልግም<br>99. መወሰን አልችልም	
244	በአሁኑ ጊዜ የእርግዝና ሁኔታዎ ምን ይመስላል?	1. ነፍሰጡር ነኝ<br>2. ነፍሰ ጡር አይደለሁም<br>3. ነፍሰጡር መሆንና አለመሆኔን አላዉቅመ	
245	እስካሁን ያሉ ልጆችን ጨምሮ በአጠቃላይ እንዲኖሩዎት የሚፈልጓቸዉ ልጆች ብዛት	…………(ቁጥር ያስቀምጡ)	

ጊዜዎትን ሰውተው ይህን ጠቃሚ መረጃ ስለሰጡኝ በጣም አመሰግናለሁ፡፡ሌላ አስተያየት ካለዎት ሊነግሩኝ ይችላሉ፡፡____
