# Supplementary material for: The role of maternal health care services as predictors of time to modern contraceptive use after childbirth in Northwest Ethiopia: Application of the shared frailty survival analysis
Source: PLoS One. 2020 Feb 4;15(2):e0228678. doi: 10.1371/journal.pone.0228678 (PMC6999900; doi:10.1371/journal.pone.0228678)
Supplement: Attachment — Submitted filename: comments 3.docx [file pone.0228678.s004.docx]

The manuscript is related to the role of maternal health care services as predictors of time to modern contraceptive use after childbirth in North West Ethiopia using Gamma distribution for shared frailty survival analysis with different distributions. The manuscript is generally well written and clearly presented. However, some minor corrections should be done.

1- In Table 1 for each variable percentages of contraceptive users and nonusers are needed.
2- For table 3, for Log-likelihood and AIC values, decimals are not needed.
3- In Table 4, the number of decimal places must be the same.
4- Information of Fig1 with Table2 are the same. One of them should be deleted.
5- Please check baseline hazard function of Weibull distribution in Line192. What is the scale parameter of the Weibull distribution?
6- The interpretations of Multivariable Survival Analysis results should be modified. For example, what do you mean “The likelihood of initiating contraceptive use for women who had four or more antenatal visits was 59 percent (aHR =1.59; 95% CI: 1.22–2.06) higher than for women who had less number of visits.”
7- There is no for some sentences reference. For e.g. “Consequently, modeling time to contraceptive use ignoring the frailty terms may lead to biased estimates of parameters and their respective standard errors.”. Please check the whole manuscript again.
