# Supplementary material for: The role of maternal health care services as predictors of time to modern contraceptive use after childbirth in Northwest Ethiopia: Application of the shared frailty survival analysis
Source: PLoS One. 2020 Feb 4;15(2):e0228678. doi: 10.1371/journal.pone.0228678 (PMC6999900; doi:10.1371/journal.pone.0228678)
Supplement: Attachment — Submitted filename: Response to Reviewers.docx [file pone.0228678.s005.docx]

**Response to Reviewers**

Dear Editor,

We are very grateful for your letter and the opportunity to improve and resubmit our manuscript. We appreciate the time and effort that you and the reviewers have dedicated to providing your valuable comments and suggestions on our manuscript. The comments and suggestions provided have been immensely helpful. We have revised the manuscript based on the comments and suggestions.

Kindly find below our point-by-point responses to the reviewers’ comments. Unless otherwise stated, all line numbers and pages are in reference to the revised manuscript.

Sincerely,

Amanu Aragaw Emiru

(On the behalf of Co-Authors)

**Comments from Reviewer 1**

**Comment** #**1:** on line 193, the index for frailty term is not correct, basing on equation specification j index represent for individual and i for cluster. For this case, frailty is taking into consideration clusters heterogeneity not individuals.

**Response:** Thank you for pointing this out; please consider this as a typo. This has now been corrected (page 9, line 193)

**Comment #2:** For tables, they should add percentage to other column as they did for total column.

**Response:** Agree. We have incorporated percentages both for contraceptive user and for nonuser columns (page 11-12, line 223).

**Comments from Reviewer 2**

**Comment** #**1:** In Table 1, for each variable percentages of contraceptive users and nonusers are needed.

**Response:** Thank you for your suggestion. We have added percentages for both contraceptive user and nonuser columns

**Comment** #2**:** For table 3, for Log-likelihood and AIC values, decimals are not needed.

**Response:** Decimals are removed as per your suggestion, thank you.

**Comment** #3**:** In Table 4, the number of decimal places must be the same.

**Response:** Done.

**Comment** #4**:** Information of Fig1 with Table2 are the same. One of them should be deleted.

**Response:** We agree, we have removed table 2 from the manuscript

**Comment** #5**:** Please check baseline hazard function of Weibull distribution in Line192. What is the scale parameter of the Weibull distribution?

**Response:** We agree that the symbols was not clear as it stands. We have slightly modified the hazard function to ease readers understand the model. In the revised version, Zi exp (β’xij) denotes the scale parameter and ρ the shape parameter.

**Comment** #6**:** The interpretations of Multivariable Survival Analysis results should be modified. For example, what do you mean “The likelihood of initiating contraceptive use for women who had four or more antenatal visits was 59 percent (aHR =1.59; 95% CI: 1.22–2.06) higher than for women who had less number of visits.”

**Response:** We are grateful for this comment. We tried to revise this section as per your comment. The revised section is highlighted on line 288-299 (page 15)

**Comment** #7**:** There is no for some sentences reference. For e.g. “Consequently, modeling time to contraceptive use ignoring the frailty terms may lead to biased estimates of parameters and their respective standard errors.” Please check the whole manuscript again.

**Response:** Thanks for this comment and suggestion. We have carefully read the manuscript and have added references as advised. The references considered are highlighted in the revised version.
